# HCFSL-UAV: Hierarchical Cluster-Head Federated Split Learning for UAV Networks

**DOI:** 10.3390/s26196331

**Published:** 2026-10-07

**Authors:** Ruomeng Yuan, Jing Fan, Hao Yu, Yu Peng, Xing Xu

**Affiliations:** 1 School of Electrical and Information Technology, Yunnan Minzu University, Kunming 650504, China; 2 Yunnan Key Laboratory of Unmanned Autonomous System, Yunnan Minzu University, Kunming 650504, China; 3 University Key Laboratory of Information and Communication on Security Backup and Recovery, Yunnan Minzu University, Kunming 650504, China

**Keywords:** UAV networks, federated split learning, dynamic model partitioning, client selection, hierarchical aggregation, edge intelligence, alternating optimization, federated learning

## Abstract

Resource constraints, time-varying links, and heterogeneous data distributions make distributed model training difficult in unmanned aerial vehicle (UAV) networks. Conventional federated learning can impose substantial computational and communication burdens on resource-constrained UAVs, whereas existing split-learning schemes commonly rely on fixed split points and static participation strategies. To address these limitations, this paper proposes HCFSL-UAV, a hierarchical cluster-head federated split learning framework for UAV networks. The framework adopts a three-layer architecture composed of a central base station, cluster-head UAVs, and member UAVs, and integrates split learning within hierarchical federated aggregation. Member UAVs are dynamically associated with cluster heads according to their own real-time 3-D spatial locations. Within each cluster, participant selection and model splitting are jointly optimized as the set of selected UAVs affects data representativeness and synchronous training latency, while the split point determines the computation, memory, and communication requirements of participating UAVs. A joint optimization problem is formulated to balance data-distribution deviation, training latency, and resource consumption, and an alternating optimization procedure is developed using greedy participant selection and a discrete split-point search. Experiments on FMNIST, CIFAR-10, and CIFAR-100 show that HCFSL-UAV achieves faster convergence and higher test accuracy than the considered federated-learning and split-learning baselines, while reducing communication and computation overhead in the reported settings. These results demonstrate the effectiveness of jointly optimizing participant selection and adaptive model partitioning in dynamic and heterogeneous UAV networks.

## 1. Introduction

With the development of sixth-generation mobile communication and edge intelligence technologies, unmanned aerial vehicle (UAV) networks have been widely deployed in various missions including environmental monitoring, emergency rescue, target tracking and topographic mapping, and have gradually become a vital component of the integrated space–air–ground intelligent system. Different from traditional terrestrial edge computing networks, UAV networks feature highly dynamic topologies, air-to-air communication links vulnerable to environmental interference, and severe heterogeneity in computing and energy resources across devices. These characteristics make it difficult for conventional centralized machine learning methods to achieve efficient deployment in such scenarios [1,2].

To protect data privacy and reduce the communication pressure caused by centralized training, federated learning (FL) has been extensively adopted in distributed UAV intelligent systems. Nevertheless, classic federated learning algorithms represented by FedAvg suffer from slow model convergence, severe accuracy degradation and poor training stability when coping with non-independent and identically distributed (non-IID) data, heterogeneous computing and energy consumption capabilities of nodes, and dynamic network topologies in UAV networks [2,3]. Meanwhile, standard federated learning imposes excessive workload and overhead on resource-constrained UAVs, failing to meet the practical training requirements of UAV networks in field environments.

Split learning splits a neural network into a client-side sub-model and a server-side sub-model. It offloads the computation of the latter network layers to edge nodes and only transmits intermediate features, which greatly reduces the local computing burden on end devices [4]. However, conventional split learning schemes are often difficult to adapt to heterogeneous device resources and dynamically changing network conditions. Recent studies have therefore investigated resource-aware and heterogeneous model splitting mechanisms [5,6]. Nevertheless, differences in node resource status and data distribution still need to be considered during collaborative training to avoid model training drift, slow convergence and unnecessary resource waste.

As the scale and heterogeneity of wireless edge devices continue to grow, clustered federated learning has been gradually integrated into federated split learning due to its advantages in handling non-IID data and improving system scalability. Current relevant studies mainly cluster edge nodes, where cluster heads undertake the parallel computation of sub-models and local aggregation within clusters during collaborative training, so as to alleviate the communication bottleneck of the central server.

However, most existing studies on clustered federated split learning are based on the assumption of static or fixed network topologies. Such static clustering mechanisms cannot be directly applied to UAV networks with highly dynamic topologies and time-varying channels. In field or three-dimensional airspace environments, the rapid movement, energy depletion of UAVs and environmental occlusion cause frequent fluctuations of air-to-air (A2A) and air-to-ground (A2G) links. Based on the above analysis, this paper proposes a framework for dynamic UAV networks, as illustrated in Figure 1. Figure 1 depicts the target aerial collaborative training scenario consisting of one central ground base station S, multiple cluster-head UAVs (CU), and numerous member UAVs (MU) distributed within the target geographical area. Member UAVs are further divided into participating member UAVs (PU) that join the current training round and non-selected member UAVs (MU). Each cluster-head UAV manages one independent cluster coverage region. Member UAVs collect local sensing data and undertake the client-side sub-model computation. Cluster-head UAVs receive intermediate feature outputs from participating member UAVs, execute the server-side part of the split neural network, and complete intra-cluster model aggregation. The central base station communicates with all cluster-head UAVs to implement global model aggregation and broadcast updated global model parameters. This work focuses on the dynamic clustering and adaptive association of cluster-head UAVs. Getting rid of the reliance on fixed network topologies, the framework fully considers the high mobility and dynamic link fluctuations of UAV networks in three-dimensional space. At the initialization phase of each training round, all member UAVs autonomously associate with corresponding cluster heads following the nearest neighbor rule based on the real-time three-dimensional Euclidean distances between member UAVs and cluster-head UAVs, so as to dynamically construct and adjust the hierarchical cluster structure. On this basis, we further establish a joint optimization model for dynamic participant selection and adaptive model splitting. Taking data distribution representativeness, training latency and resource consumption as optimization objectives, the model characterizes the coupling relationship between the two strategies.

The main contributions of this paper are as follows:

We propose HCFSL-UAV, a hierarchical cluster-head federated split learning framework for dynamic UAV networks. The positions of member UAVs and their cluster-head associations are updated in each training round. These updates are reflected in the inter-UAV distances and channel conditions used for communication.

We jointly consider participant selection and model splitting. The UAVs selected for training affect the data distribution and resource conditions within a cluster, while the split point affects its communication and computation costs. We therefore determine the participant set and split point together, rather than fixing one before optimizing the other.

We use alternating optimization to solve the resulting discrete problem. In each iteration, a greedy procedure selects the participating UAVs, and a search over candidate split points determines where to partition the model. This avoids evaluating every possible combination of participants and split points.

We evaluate HCFSL-UAV on FMNIST, CIFAR-10, and CIFAR-100. Comparisons with FedAvg, FedProx, and split-learning baselines report test accuracy, convergence, communication cost, and computational workload.

## 2. Related Work

Focusing on the core technical direction of this paper, this section reviews the mainstream research paradigms and representative achievements in the field of UAV federated intelligence, and sorts out the relevant state-of-the-art research, which lays a theoretical foundation for the proposed HCFSL-UAV framework.

### 2.1. UAV-Assisted Federated Learning

Existing studies mainly focus on resource collaborative optimization for UAV-assisted wireless and edge intelligence systems, including UAV trajectory planning, communication resource allocation, terminal scheduling and energy consumption optimization. Reference [7] jointly optimizes UAV flight trajectory and transmits power to improve system data transmission efficiency and communication reliability. References [8,9,10] jointly consider UAV position or trajectory, computation task partitioning, scheduling and communication resources to reduce system energy consumption and processing latency. Reference [11] investigates UAV-assisted wireless powered cooperative mobile edge computing through joint offloading, CPU control and trajectory optimization, while Reference [12] studies UAV deployment optimization for UAV-assisted cellular networks.

With the integration of federated learning into UAV networks, resource allocation and collaborative training have received increasing attention. Reference [13] investigates federated learning with UAV swarms and jointly optimizes power allocation and UAV scheduling. Reference [14] jointly optimizes federated learning training and resource allocation in UAV swarms. Reference [15] further develops a hierarchical nested personalized federated learning framework for UAV-assisted multi-tier networks. In addition, Reference [16] investigates an energy-efficient 6G federated learning-enabled UAV deployment scheme.

Several studies further focus on communication and energy efficiency in UAV-assisted federated learning. Reference [17] investigates UAV communications for sustainable federated learning. Reference [18] considers energy-efficient federated learning over UAV-enabled wireless powered communications. Reference [19] studies federated learning via unmanned aerial vehicles, while Reference [20] jointly optimizes UAV trajectory, device scheduling and resource management to improve the energy efficiency of UAV-assisted federated learning.

Although existing research on UAV-assisted federated learning has made substantial progress in resource optimization and collaborative training, most of these studies are developed based on conventional federated learning frameworks. In UAV scenarios with device heterogeneity, resource constraints and energy sensitivity, these methods may still cause high terminal computation load and communication overhead, which limits their applicability to resource-constrained UAV edge intelligence systems.

### 2.2. Split Learning

To reduce the training burden of conventional federated learning on resource-constrained edge devices, split learning (SL) divides a deep neural network into a client-side sub-model and a server-side sub-model. Only intermediate activation values and gradient information are exchanged at the splitting layer, thereby reducing the computing and storage overhead on the terminal side [4]. Early studies introduced distributed neural network training through model partitioning across multiple agents [21,22], laying the foundation for subsequent split learning research.

On this basis, researchers have integrated federated learning and split learning to combine the parallel training capability of federated learning with the lightweight client-side computation of split learning. Typical methods include SplitFed [23] and SplitMix [24]. SplitFed integrates model splitting and federated aggregation to alleviate client-side training pressure while maintaining distributed parallel training capability [23]. SplitMix enables heterogeneous model configurations for distributed clients and improves system adaptability to heterogeneous devices [24]. In addition, Reference [25] proposed a hybrid split and federated learning approach for wireless distributed learning, combining the advantages of the two distributed training paradigms.

With the increasing heterogeneity of edge devices, adaptive model splitting and resource-aware split learning mechanisms have attracted increasing attention. Reference [5] proposes the ARES framework, which adaptively determines model partitioning according to resource conditions in Internet-of-Things environments. Reference [6] investigates split federated learning over heterogeneous edge devices and allows clients with heterogeneous resources to adopt different model splitting configurations.

Nevertheless, most existing federated split learning studies are designed for relatively stable client-server associations and cannot directly adapt to dynamically changing computing resources and communication links in UAV networks. In addition, participant selection and model splitting are generally optimized separately, while the coupling relationship between the two strategies has not been sufficiently considered.

### 2.3. Client Selection in Distributed Learning

In wireless edge learning systems, not all terminals can stably participate in model updating in each training round because of heterogeneous computing resources and time-varying wireless links. Therefore, selecting participating nodes with appropriate resource conditions and useful training data has become an important research issue in distributed learning.

Existing client selection strategies mainly consider communication conditions, resource availability and learning contribution. Reference [26] proposes a client selection strategy for federated learning with heterogeneous resources in mobile edge environments. Reference [27] constructs a joint learning and communication framework for federated learning over wireless networks. Reference [28] investigates reliable, federated learning for mobile networks by considering the reliability of participating clients.

Meanwhile, several studies consider the importance of local model updates during client scheduling. Reference [29] investigates update-aware device scheduling and analyzes its convergence performance in wireless federated learning. Reference [30] further proposes an importance- and channel-aware scheduling mechanism that jointly considers model update importance and wireless channel conditions.

However, resource states, communication conditions and learning contributions may be difficult to accurately estimate before training. To address client scheduling under uncertain environments, multi-armed-bandit (MAB) methods have been introduced into federated learning. Reference [31] proposes bandit-based communication-efficient client selection strategies for federated learning. Reference [32] develops an MAB-based client selection strategy for mobile networks with uncertain resources. Reference [33] further proposes a multi-armed-bandit-based client scheduling method for federated learning.

Despite the progress of existing client selection methods, most of them are designed for conventional federated learning and do not consider the characteristics of federated split learning. In heterogeneous UAV scenarios, device resources, communication conditions and model splitting positions jointly affect training efficiency and model performance. Therefore, the joint optimization of dynamic participant selection and adaptive model splitting remains an important problem to be addressed.

## 3. System Model and Problem Formulation

### 3.1. Network Architecture

To accommodate the characteristics of UAV networks in field data collection scenarios, including constrained computational resources, dynamic link conditions, and heterogeneous data distributions, we consider a hierarchical cluster-head federated split learning framework, termed HCFSL-UAV, as illustrated in Figure 2. The upper part illustrates the three-tier network architecture, including the central base station, cluster-head UAVs and member UAVs, as well as the interaction flows among different network entities. The lower part depicts the alternating-optimization workflow, which consists of two coupled sub-modules: dynamic member-UAV selection and adaptive model splitting. In the dynamic member-UAV selection module, candidate UAVs are generated firstly, followed by scoring-evaluation and greedy participant selection under resource and data-representativeness constraints. In the adaptive model-splitting module, feasible split-point candidates are filtered by resource constraints, and the optimal split point is obtained through cost evaluation.

Different clusters may choose different split points in the same training round. Before training, the global model is partitioned separately for each cluster while retaining the same layer order and parameter shapes. For each cluster, participating member UAVs update the layers before its split point, and the cluster head updates the remaining layers using the uploaded activations. After training, the member-side and cluster-head-side parameters are mapped to their corresponding layers in the full global model. The base station aggregates the updates layer by layer, weighting each member-UAV update by its recorded participating sample count and each cluster-head update by the total recorded participating sample count of that cluster. Thus, different split points determine where a layer is trained, but do not require averaging sub-models of different shapes.

The two sub-modules iterate alternately to solve the joint optimization problem. The network consists of one central base station, N={1,2,…,N} member UAVs, and K={1,2,…,K} cluster-head UAVs, which collaboratively train a shared global model through hierarchical federated split learning.

The member-UAV and cluster-head index sets are

(1) $$U_{\mathrm{MU}}=\left\{{\mathrm{MU}}_{i}|i\in N\right\},U_{\mathrm{CU}}=\left\{{\mathrm{CU}}_{j}|j\in K\right\}$$

Let *t* denote the global training-round index. Let $q_{i}(t)={\left[x_{i}(t),y_{i}(t),z_{i}(t)\right]}^{T}$ and $q_{j}(t)={\left[x_{j}(t),y_{j}(t),z_{j}(t)\right]}^{T}$ denote the current three-dimensional positions of member UAV i∈N and cluster-head UAV j∈K, respectively. Their Euclidean distance is

(2) $$d_{ij}(t)=\;∥q_{i}(t)-q_{j}(t){∥}_{2}\;=\sqrt{{\left(x_{i}(t)-x_{j}(t)\right)}^{2}+{\left(y_{i}(t)-y_{j}(t)\right)}^{2}+{\left(z_{i}(t)-z_{j}(t)\right)}^{2}}$$

To represent UAV mobility without introducing excessive modeling complexity, the position of UAV *i* is updated between two consecutive global training rounds according to

(2a) $$q_{i}(t+1)=q_{i}(t)+v_{i}(t)\Delta T$$

where $v_{i}(t)$ denotes the three-dimensional velocity of UAV *i*, and ΔT denotes the duration of one global training round. When a UAV reaches the boundary of the considered flight area, a new flight direction is generated while its speed remains within the prescribed range. The cluster-head UAVs can follow the same mobility rule or remain quasi-stationary during the considered training period.

At the beginning of each global training round, every member UAV associates with its nearest cluster-head UAV according to

(3a) $$j_{i}^{*}(t)=\underset{j\in K}{\arg\min}d_{ij}(t)$$

and the set of member UAVs served by cluster-head UAV j is

(3b) $$U_{j}(t)=\{i\in N|j_{i}^{⋆}(t)=j\}$$

Member UAVs collect and store local data and execute the client-side layers of the split model. Cluster-head UAVs receive intermediate activations, execute the remaining layers, and perform intra-cluster aggregation. The central base station performs global aggregation and broadcasts the updated global model. The following formulation focuses on the two coupled cluster-level decisions: the participating member set $S_{j}$ and the split point $l_{j}$.

### 3.2. Delay and Cost Modeling

Consider member UAV *i* and its associated cluster-head UAV *j* in global training round t. Bithas et al. [34] investigated the effects of mobility and shadowing on UAV-to-ground communication channels. For the member-to-cluster-head air-to-air links considered here, we adopt a simplified time-varying channel model that incorporates the instantaneous three-dimensional distance, distance-dependent path loss, log-normal shadowing, and Rician small-scale fading. The effective channel power gain is

(4a) $$g_{ij}(t)=\beta_{0}{\left(\frac{d_{ij}(t)}{d_{0}}\right)}^{-\eta }10^{-\frac{X_{ij}(t)}{10}}{\left|h_{ij}(t)\right|}^{2}$$

Here, $\beta_{0}$ is the channel power gain at reference distance $d_{0}$, η is the path-loss exponent, and Xij(t) is the shadowing term in dB. The shadowing term is modeled as

(4b) $$X_{ij}(t)∼N\left(0,\sigma_{sh}^{2}\right)$$

where $\sigma_{\mathrm{sh}}$ is the shadowing standard deviation. To represent the line-of-sight and scattered components of an air-to-air link, the small-scale fading coefficient is modeled as

(4c) $$h_{ij}(t)=\sqrt{\frac{K_{Ric}}{K_{Ric}+1}}e^{j\phi_{ij}(t)}+\sqrt{\frac{1}{K_{Ric}+1}}\underset{ij}{\tilde{h}}(t)$$

where $K_{Ric}$ denotes the Rician factor, $\phi_{ij}(t)$ is the phase of the line-of-sight component, and $\underset{ij}{\tilde{h}}(t)$ represents the scattered component satisfying

(4d) $$\underset{ij}{\tilde{h}}(t)∼CN(0,1)$$

The channel is assumed to remain quasi-static within one global training round, and to change between rounds. Thus, $g_{ij}(t)$ is the effective channel power gain in round t, not a fixed long-term average gain. It is updated at the beginning of each round using the current UAV positions and the corresponding path-loss, shadowing, and fading terms.

Using the current-round channel power gain $g_{ij}(t)$ defined in (4a), let $p_{i}^{UL}$ denote the uplink transmit power of member UAV *i* and $p_{ij}^{DL}$ the downlink transmit power allocated by cluster-head UAV *j* to member UAV *i*. The corresponding uplink and downlink bandwidths are $W_{ij}^{UL}$ and $W_{ij}^{DL}$, respectively. $N_{0}$ denotes the noise power spectral density. Assuming channel reciprocity within a training round, the uplink and downlink transmission rates are given by (5a) and (5b), respectively.

(5a) $$R_{ij}^{UL}(t)=W_{ij}^{UL}\log_{2}\left(1+\frac{p_{i}^{UL}g_{ij}(t)}{N_{0}W_{ij}^{UL}}\right)$$

(5b) $$R_{ij}^{DL}(t)=W_{ij}^{DL}\log_{2}\left(1+\frac{p_{ij}^{DL}g_{ij}(t)}{N_{0}W_{ij}^{DL}}\right)$$

For split point $l_{j}$, communication between member UAV i and cluster-head UAV j includes four components: downloading the client-side sub-model, uploading the intermediate activations, downloading the corresponding gradients, and uploading the updated client-side sub-model. Let $\varpi_{l_{j}}^{D}$ denote the client-side sub-model size in bits, and let $\varpi_{l_{j}}^{S}$ and $\varpi_{l_{j}}^{G}$ denote the activation and gradient sizes per sample, respectively. For mini-batch size $B_{i}$, the four transmission delays are

(6a) $$\tau_{ij}^{mdl,DL}\left(l_{j}\right)=\frac{\varpi_{l_{j}}^{D}}{R_{ij}^{DL}(t)}$$

(6b) $$\tau_{ij}^{act,UL}\left(l_{j}\right)=\frac{B_{i}\varpi_{l_{j}}^{S}}{R_{ij}^{UL}(t)}$$

(6c) $$\tau_{ij}^{grad,DL}\left(l_{j}\right)=\frac{B_{i}\varpi_{l_{j}}^{G}}{R_{ij}^{DL}(t)}$$

(6d) $$\tau_{ij}^{mdl,UL}\left(l_{j}\right)=\frac{\varpi_{l_{j}}^{D}}{R_{ij}^{UL}(t)}$$

Let $f_{i}$ denote the processor frequency in cycles/s, $\rho_{i}$ the number of floating-point operations executed per CPU cycle, and $C_{l_{j}}^{FP}$ and $C_{l_{j}}^{BP}$ the forward- and backward-propagation costs per sample in FLOPs/sample. The corresponding computation delays are

(7a) $$\tau_{i}^{FP}\left(l_{j}\right)=\frac{B_{i}C_{l_{j}}^{FP}}{f_{i}\rho_{i}}$$

(7b) $$\tau_{i}^{BP}\left(l_{j}\right)=\frac{B_{i}C_{l_{j}}^{BP}}{f_{i}\rho_{i}}$$

For compactness, define the total communication delay as

(8) $$\tau_{ij}^{comm}\left(l_{j}\right)≜\tau_{ij}^{mdl,DL}\left(l_{j}\right)+\tau_{ij}^{act,UL}\left(l_{j}\right)+\tau_{ij}^{grad,DL}\left(l_{j}\right)+\tau_{ij}^{mdl,UL}\left(l_{j}\right)$$

The member-side latency is then

(9) $$T_{ij}\left(l_{j}\right)=\tau_{ij}^{comm}\left(l_{j}\right)+\tau_{i}^{FP}\left(l_{j}\right)+\tau_{i}^{BP}\left(l_{j}\right)$$

Let $p_{i}^{RX}$ denote the receive power of member UAV i. The communication energy can be written compactly as

(10) $$E_{ij}^{comm}\left(l_{j}\right)=p_{i}^{RX}\left(\tau_{ij}^{mdl,DL}+\tau_{ij}^{grad,DL}\right)+p_{i}^{UL}\left(\tau_{ij}^{act,UL}+\tau_{ij}^{mdl,UL}\right)$$

where all delay terms in (10) are evaluated at $l_{j}$. Let $\kappa_{i}$ denote the effective switched-capacitance coefficient of UAV i. Under the dynamic CPU power model, the computation energy is

(11) $$E_{i}^{comp}\left(l_{j}\right)=\frac{\kappa_{i}B_{i}\left(C_{l_{j}}^{FP}+C_{l_{j}}^{BP}\right)f_{i}^{2}}{\rho_{i}}$$

Because the communication component depends on the associated cluster head, the resource cost is indexed by both i and j:

(12) $$c_{ij}\left(l_{j}\right)=\lambda_{1}E_{i}^{comp}\left(l_{j}\right)+\lambda_{2}E_{ij}^{comm}\left(l_{j}\right)$$

where $\lambda_{1},\lambda_{2}\geq 0$ and $\lambda_{1}+\lambda_{2}=1$.

Unless otherwise stated, these expressions do not include computation performed by the cluster-head UAVs, global-model aggregation at the base station, or communication between the cluster-head UAVs and the base station. These excluded terms are independent of the member-side feasibility constraints considered in the participant-selection and model-partitioning problems.

### 3.3. Dynamic Member Feasibility and Data Representation

After the UAV positions, channel conditions, and cluster associations are updated at the beginning of global training round t, not every UAV in the current cluster $U_{j}(t)$ is eligible to participate. Eligibility depends on residual energy, available memory, and the member-side latency calculated using the current-round transmission rates. In addition, because the local datasets are non-IID, the selected set should retain the label distribution of the complete current cluster as closely as possible. For notational simplicity, the global training-round index t is omitted from the subsequent expressions. Thus, $U_{j}$ represents the current-round cluster membership set $U_{j}(t)$, and all communication-related quantities are evaluated using the current-round channel gain and transmission rates in (4a)–(5b).

Let $n_{i}$ denote the local dataset size of UAV i. Its empirical label-distribution vector is $p_{i}=\left[p_{i,1},p_{i,2},\ldots ,p_{i,C}\right]$, where $p_{i,c}$ is the proportion of class-c samples and $\sum_{c=1}^{C}p_{i,c}=1$. The reference distribution of cluster j is

(13) $$p_{j}^{ref}=\sum_{i\in U_{j}}\frac{n_{i}}{\sum_{k\in U_{j}}n_{k}}p_{i}$$

For a nonempty participating set $S_{j}\subseteq U_{j}$, its aggregated label distribution is

(14) $$p_{S_{j}}=\sum_{i\in S_{j}}\frac{n_{i}}{\sum_{k\in S_{j}}n_{k}}p_{i}$$

The representation loss is defined by the Jensen–Shannon divergence:

(15) $$R_{j}\left(S_{j}\right)=D_{JS}\left(p_{S_{j}}\;∥\;p_{j}^{ref}\right)$$

A smaller $R_{j}\left(S_{j}\right)$ indicates better statistical representativeness of the selected set.

For a given split point $l_{j}$, the feasible candidate set is

(16) $${\tilde{U}}_{j}\left(l_{j}\right)=\{i\in U_{j}|E_{i}^{res}\geq E_{i}^{min},\;M_{i}^{avail}\geq M_{i}\left(l_{j}\right),\;T_{ij}\left(l_{j}\right)\leq T^{max}\}$$

Here, $E_{i}^{res}$ denotes residual energy, $E_{i}^{min}$ the prescribed residual-energy threshold for participation, $M_{i}^{avail}$ available memory, $M_{i}\left(l_{j}\right)$ the memory requirement at split point $l_{j}$, and $T^{max}$ the maximum tolerable member-side latency.

To combine latency and resource cost in the same objective, both terms are normalized. The normalized latency is

(17) $${\hat{T}}_{ij}\left(l_{j}\right)=\frac{T_{ij}\left(l_{j}\right)}{T^{max}}$$

and the normalized resource cost is

(18) $$\begin{array}{cc} {\hat{c}}_{ij}\left(l_{j}\right)=\frac{c_{ij}\left(l_{j}\right)}{c_{j}^{ref}}, & c_{j}^{ref}=\underset{i\in U_{j},\;l\in L}{max}c_{ij}\left(l\right) \end{array}$$

The reference value $c_{j}^{ref}$ is fixed during the current optimization round so that costs obtained under different candidate split points are compared on the same scale. For a nonempty set $S_{j}$, define the average normalized latency and resource cost as ${\bar{T}}_{j}\left(S_{j},l\right)≜{\left|S_{j}\right|}^{-1}\sum_{i\in S_{j}}{\hat{T}}_{ij}\left(l\right)$ and ${\bar{c}}_{j}\left(S_{j},l\right)≜{\left|S_{j}\right|}^{-1}\sum_{i\in S_{j}}{\hat{c}}_{ij}\left(l\right)$, respectively.

For a nonempty participating set $S_{j}$ under split point l, the participant-selection cost is defined as

(19) $$F_{j}\left(S_{j},l\right)=\omega_{1}R_{j}\left(S_{j}\right)+\omega_{2}{\bar{T}}_{j}\left(S_{j},l\right)+\omega_{3}{\bar{c}}_{j}\left(S_{j},l\right)$$

The weights satisfy $\omega_{1},\omega_{2},\omega_{3}\geq 0$ and $\omega_{1}+\omega_{2}+\omega_{3}=1$.

### 3.4. Adaptive Model Partitioning Feasibility Modeling

Let L denote the candidate split-point set. A deeper split point generally increases member-side computation and memory demand, whereas a shallower split point can increase activation and gradient transmission. Because the transmission rates vary across global training rounds, the feasible and preferred split points may also change with the current UAV positions and channel conditions. For a given participating set $S_{j}$, the feasible split-point set is

(20) $$L_{j}^{fea}\left(S_{j}\right)=\{l\in L|M_{i}^{avail}\geq M_{i}\left(l\right),\;E_{i}^{res}\geq E_{i}^{min},\;T_{ij}\left(l\right)\leq T^{max},\;\forall i\in S_{j}\}$$

Although the global training-round index is omitted, the feasible split-point set in (20) is reconstructed in every global training round using the current-round transmission rates and resource states.

For synchronous intra-cluster training, the slowest member determines the member-side completion time. Using the normalized quantities introduced above, the split-point cost is

(21) $$\Psi_{j}\left(l;S_{j}\right)=\mu_{1}\underset{i\in S_{j}}{max}{\hat{T}}_{ij}\left(l\right)+\mu_{2}{\bar{T}}_{j}\left(S_{j},l\right)+\mu_{3}{\bar{c}}_{j}\left(S_{j},l\right).$$

The weights satisfy $\mu_{1},\mu_{2},\mu_{3}\geq 0$ and $\mu_{1}+\mu_{2}+\mu_{3}=1$. The first term captures the straggler effect, while the remaining terms represent average normalized latency and resource cost.

For a fixed participating set $S_{j}$, the preferred split point is

(22) $$l_{j}^{*}=\underset{l\in L_{j}^{fea}\left(S_{j}\right)}{\arg\min}\Psi_{j}\left(l;S_{j}\right)$$

Because $L_{j}^{fea}\left(S_{j}\right)$ is finite, (22) is solved by one-dimensional discrete search.

### 3.5. Problem Formulation

The participating set $S_{j}$ determines the data distribution and the UAVs contributing to synchronous training, whereas the split point $l_{j}$ determines client-side computation, memory demand, and A2A communication load. The two decisions are therefore coupled through both feasibility and cost.

To ensure that participant selection and model partitioning optimize the same quantity, define the cluster-level joint cost as $J_{j}\left(S_{j},l\right)=\alpha F_{j}\left(S_{j},l\right)+\left(1-\alpha \right)\Psi_{j}\left(l;S_{j}\right)$, $\alpha \in \left[0,1\right]$. Since $F_{j}$ and $\Psi_{j}$ are dimensionless, α directly controls their relative contribution. The joint problem is

(23) $$\begin{array}{c} \underset{{\left\{S_{j}\right\}}_{j=1}^{K},{\left\{l_{j}\right\}}_{j=1}^{K}}{min}J=\sum_{j=1}^{K}J_{j}\left(S_{j},l_{j}\right)\\ \begin{array}{cc} s.t. & \begin{array}{ll} S_{j}\subseteq U_{j}, & \forall j\in K\\ N_{j}^{min}\leq \left|S_{j}\right|\leq N_{j}^{max}, & \forall j\in K\\ l_{j}\in L, & \forall j\in K \end{array} \end{array} \end{array}$$

## 4. Alternating Optimization Algorithm

As formulated in (23), the participating member set $S_{j}$ and the model split point $l_{j}$ are coupled discrete variables. Direct joint enumeration requires evaluating a large number of participant-set and split-point combinations. We therefore decompose the problem into two subproblems and update them alternately. In each alternating-optimization (AO) iteration, the member set is updated for a fixed split point, after which the split point is optimized for the resulting member set. Both subproblems use the same cluster-level joint cost $J_{j}\left(S_{j},l_{j}\right)$ defined in the preceding section.

To distinguish the AO iteration from the global training-round index, r denotes the AO iteration index throughout this section.

### 4.1. Dynamic Member UAV Selection Algorithm

For a fixed split point $l_{j}$, the member-selection subproblem for cluster j is

(24) $$\underset{S_{j}\subseteq {\tilde{U}}_{j}\left(l_{j}\right)}{min}J_{j}\left(S_{j},l_{j}\right),\;\;N_{j}^{min}\leq \left|S_{j}\right|\leq N_{j}^{max}$$

Here, ${\tilde{U}}_{j}\left(l_{j}\right)$ is the feasible candidate set defined in (16). If $\left|{\tilde{U}}_{j}\left(l_{j}\right)\right|<N_{j}^{min}$, the current split point cannot support the required minimum number of participants and is treated as infeasible for the member-selection subproblem.

Because $J_{j}\left(S_{j},l_{j}\right)$ contains statistics and averages over $S_{j}$, it is not defined for an empty set. Therefore, the greedy procedure is initialized with the feasible singleton having the smallest joint cost,

(25) $$i_{0}^{*}=arg\underset{i\in {\tilde{U}}_{j}\left(l_{j}\right)}{min}J_{j}\left(\{i\},l_{j}\right),\;\;S_{j}^{init}=\{i_{0}^{*}\}$$

For the current nonempty selected set $S_{j}$, let $S_{j}^{\prime }$ denote the set obtained by adding candidate UAV i to $S_{j}$. The marginal cost reduction for $i\in {\tilde{U}}_{j}\left(l_{j}\right)$ with $i\notin S_{j}$ is defined as

(26) $$\Gamma_{j}\left(i|S_{j},l_{j}\right)=J_{j}\left(S_{j},l_{j}\right)-J_{j}\left(S_{j}^{\prime },l_{j}\right)$$

A larger $\Gamma_{j}$ indicates a larger reduction in the joint cluster cost. The next candidate is selected as

(27) $$i^{*}=arg\underset{\begin{array}{c} i\in {\tilde{U}}_{j}\left(l_{j}\right)\\ i\notin S_{j} \end{array}}{max}\Gamma_{j}\left(i|S_{j},l_{j}\right)$$

Before the minimum participation size is reached, the candidate with the largest marginal cost reduction is added even when its marginal reduction is non-positive, because the constraint $\left|S_{j}\right|\geq N_{j}^{min}$ must first be satisfied. Once $N_{j}^{min}$ participants have been selected, the expansion continues only when

(28) $$\Gamma_{j}\left(i^{*}|S_{j},l_{j}\right)>0$$

and $\left|S_{j}\right|<N_{j}^{max}$. The selected set is then updated as

(29) $$S_{j}\leftarrow S_{j}\cup \{i^{*}\}$$

The expansion terminates when $N_{j}^{max}$ participants have been selected or no additional UAV can reduce the joint cost after the minimum participation requirement has been satisfied. The resulting set is denoted by ${\hat{S}}_{j}$.

Computational Complexity Analysis. Let $n_{j}$ be an upper bound on the size of the feasible candidate set constructed by Algorithm 1 for cluster j during alternating optimization, $N_{j}^{max}$ the maximum number of selected UAVs, C the number of data classes, and L=|L| the number of candidate split points. Algorithm 1 performs at most $N_{j}^{max}$ greedy expansion steps. At each step, it evaluates the marginal costs of at most $n_{j}$ remaining candidates. Evaluating the data-distribution term involves C classes, while updating the set-dependent resource statistics requires at most $O(N_{j}^{max})$ operations. Therefore, an upper bound on the complexity of participant selection in cluster j is $O\left(N_{j}^{max}n_{j}(C+N_{j}^{max})\right)$.
**Algorithm 1** Dynamic Member UAV Selection**Input:** Cluster member set *𝒰_j_*; current split point *l_j_*; participant limits $N_{j}^{min}$ and $N_{j}^{max}$**Output:** Candidate participating set ${\hat{S}}_{j}$1:Construct ${\tilde{U}}_{j}$ according to (16).2:**if** |${\tilde{U}}_{j}$| < $N_{j}^{min}$ **then**3:**return** infeasible4:**end if**5:Select the initial UAV $i_{0}^{*}$ according to (25).6:*S_j_* ← {$i_{0}^{*}$}7:**while** |*S_j_*| < $N_{j}^{max}$ **do**8:**if** 
${\tilde{U}}_{j}\setminus S_{j}=Ø$ **then**
9:**Break**10:**end if**11:Compute Γ*_j_*(*i*|*S_j_*, *l_j_*) for all *i* ∈ ${\tilde{U}}_{j}$\*S_j_* according to (26).12:Select $i^{*}$ according to (27).13:**if** |*S_j_*| < $N_{j}^{min}$ **then**14:*S_j_* ← *S_j_* ∪ {$i^{*}$}15:**else if** Γ*_j_*($i^{*}$| *S_j_*, *l_j_*) > 0 **then**16:*S_j_* ← *S_j_* ∪ {$i^{*}$}17:**else**18:**break**19:**end if**20:**end while**21:**return** ${\hat{S}}_{j}$ ← *S_j_*

For a fixed participating set, Algorithm 2 examines L candidate split points and evaluates at most $N_{j}^{max}$ participating UAVs at each split point. Its complexity is therefore $O(LN_{j}^{max})$.
**Algorithm 2** Adaptive Model Partitioning**Input:** Participating set *S_j_*; candidate split-point set *ℒ***Output:** Selected split point l_j_^*^  1: *J*_best_ ← +∞  2: l_j_^*^ ← null  3: **for each** *l* ∈ ℒ **do**  4: **if** *l* ∉ *ℒ_j_*^fea^(*S_j_*) **then**  5: **continue**  6: **end if**  7: Evaluate *J_j_*(*S_j_*, *l*).  8: **if** *J_j_*(*S_j_*, *l*) < *J*_best_ **then**  9: *J*_best_ ← *J_j_*(*S_j_*, *l*) 10: l_j_^*^ ← *l* 11: **end if** 12: **end for** 13: **return** l_j_^*^

For $R_{max}$ alternating-optimization iterations across K clusters, the overall complexity is bounded by $R_{max}\sum_{j=1}^{K}\left[N_{j}^{max}n_{j}(C+N_{j}^{max})+LN_{j}^{max}\right]$.

By comparison, exhaustive participant selection evaluates $\sum_{k=N_{j}^{min}}^{N_{j}^{max}}\left(\frac{n_{j}}{k}\right)$ candidate subsets in cluster j for each split point. This number can grow exponentially with $n_{j}$ when the permitted subset sizes increase with the cluster size. The proposed greedy procedure has polynomial complexity and is therefore more suitable for clusters containing a relatively large number of UAVs.

### 4.2. Adaptive Model Partitioning Algorithm

For a fixed participating set $S_{j}$, the split-point subproblem is

(30) $$\underset{l\in L_{j}^{fea}\left(S_{j}\right)}{min}J_{j}\left(S_{j},l\right)$$

The feasible split-point set $L_{j}^{fea}\left(S_{j}\right)$ is defined in (20). Since the candidate split-point set L is finite, each candidate split point can be checked directly against the feasibility conditions. For each feasible l, the normalized latency and resource cost are evaluated using (17)–(18), followed by $F_{j}\left(S_{j},l\right)$ from (19), $\Psi_{j}\left(l;S_{j}\right)$ from (21), and the joint cost $J_{j}\left(S_{j},l\right)$ defined before (23). The feasible split point with the smallest joint cost is selected.

If $L_{j}^{fea}\left(S_{j}\right)=⌀$, the current participating set is infeasible for model partitioning. In the AO procedure, such a candidate member set is not accepted; the previous feasible state is retained.

### 4.3. Iterative Execution of Alternating Optimization

Let $S_{j}^{\left(r\right)}$ and $l_{j}^{\left(r\right)}$ denote the accepted participating set and split point of cluster j at AO iteration r, respectively. For a fixed split point, the ideal member-set update is

(31) $$S_{j}^{\left(r+1\right)}\in arg\underset{\begin{array}{c} S_{j}\subseteq {\tilde{U}}_{j}\left(l_{j}^{\left(r\right)}\right)\\ N_{j}^{min}\leq \left|S_{j}\right|\leq N_{j}^{max} \end{array}}{min}J_{j}\left(S_{j},l_{j}^{\left(r\right)}\right)$$

for which Algorithm 1 provides a greedy candidate solution. Let this candidate be ${\hat{S}}_{j}^{\left(r+1\right)}$. To prevent an AO iteration from increasing the joint objective, the candidate is accepted only when

(32) $$J_{j}\left({\hat{S}}_{j}^{\left(r+1\right)},l_{j}^{\left(r\right)}\right)<J_{j}\left(S_{j}^{\left(r\right)},l_{j}^{\left(r\right)}\right)$$

Otherwise, $S_{j}^{\left(r+1\right)}=S_{j}^{\left(r\right)}$. This acceptance rule also avoids cycling among equal-cost member sets.

For the accepted member set, the split point is updated by the exact discrete search

(33) $$l_{j}^{\left(r+1\right)}=arg\underset{l\in L_{j}^{fea}\left(S_{j}^{\left(r+1\right)}\right)}{min}J_{j}\;\left(S_{j}^{\left(r+1\right)},l\right)$$

When several split points attain the same minimum value, the current split point is retained if it is among the minimizers; otherwise, the smallest-index minimizer is selected. The global objective at AO iteration r is

(34) $$J^{\left(r\right)}=\sum_{j=1}^{K}J_{j}\left(S_{j}^{\left(r\right)},l_{j}^{\left(r\right)}\right)$$

With the member-set acceptance rule and the exact split-point update, the accepted objective sequence is non-increasing. Since both decision variables take values in finite discrete sets, the procedure reaches a fixed feasible state after a finite number of accepted state changes under deterministic tie breaking. This statement concerns convergence of the proposed alternating procedure and does not imply global optimality of (23).

The AO procedure terminates when

(35) $$\left|J^{\left(r+1\right)}-J^{\left(r\right)}\right|<\varepsilon ,$$

where ε>0 denotes the stopping tolerance. The procedure also terminates when the decision variables remain unchanged or the maximum number of AO iterations $R_{max}$ is reached.

The output is a feasible fixed-point solution for the proposed AO procedure. Because the member-selection subproblem is solved by a greedy heuristic rather than exhaustive enumeration, no claim of global optimality is made. The complete AO-based joint decision procedure is summarized in Algorithm 3.
**Algorithm 3** AO-Based Joint Decision Algorithm**Input:** Cluster member sets {*𝒰_j_*}_j=1_^K^; split-point set *ℒ*; maximum AO iterations *R*_max_;  convergence threshold *ε***Output:** Participating sets {S_j_^*^}_j=1_^K^; split points {l_j_^*^}_j=1_^K^  1: Initialize a feasible split point *l_j_*^(0)^ for each *j* ∈ 𝒦.  2: Obtain an initial feasible set *S_j_*^(0)^ using Algorithm 1.  3: Compute *J*^(0)^ according to (34).  4: *r* ← 0  5: **repeat**
  6: **for each** *j* ∈ 𝒦 **do**  7: Obtain ${\hat{S}}_{j}^{\left(r+1\right)}$ using Algorithm 1 with fixed *l_j_*^(r)^.  8: **if** the candidate is feasible and *J_j_*(${\hat{S}}_{j}^{\left(r+1\right)}$, *l_j_*^(r)^) < *J_j_*(*S_j_*^(r)^, *l_j_*^(r)^) **then**  9: *S_j_*^(r+1)^ ← ${\hat{S}}_{j}^{\left(r+1\right)}$ 10: **Else**
 11: *S_j_*^(r+1)^ ← *S_j_*^(r)^ 12: **end if**
 13: Update *l_j_*^(r+1)^ using Algorithm 2 with fixed *S_j_*^(r+1)^. 14: **end for**
 15: Compute *J*^(r+1)^ according to (34). 16: *r* ← *r* + 1 17: **until** (35) is satisfied, the decisions remain unchanged, or *r* ≥ *R*_max_ 18: S_j_^*^ ← *S_j_*^(r)^, ∀ *j* ∈ 𝒦 19: l_j_^*^ ← *l_j_*^(r)^, ∀ *j* ∈ 𝒦 20: **return** final participating sets and split points.

## 5. Experiments and Numerical Results

To evaluate HCFSL-UAV, simulations are conducted using three image-classification datasets and several federated and split-learning baselines. Performance is examined in terms of test accuracy, convergence behavior, communication overhead, computation workload, and the effects of participant selection and model partitioning.

### 5.1. Experiment Environment

The experiments are conducted on a computing platform equipped with an Intel Core i9-13900HX CPU and an NVIDIA RTX 4060 GPU under Windows 11. The implementation uses Python 3.9 and PyTorch 1.13.

The simulated UAV network contains one central base station, three cluster-head UAVs, and 100 member UAVs distributed within a circular area of radius 500 m. Member UAVs fly at altitudes of 80–120 m with a speed of 10 m/s, while the cluster-head UAVs maintain an altitude of 120 m. The main simulation parameters are summarized in Table 1.

Three benchmark datasets are used to evaluate the model-partitioning mechanism under different task complexities. FMNIST contains 10 classes of 28 × 28 grayscale images. CIFAR-10 contains 10 classes of 32 × 32 color images, while CIFAR-100 contains 100 fine-grained classes. FMNIST uses a lightweight convolutional neural network with two convolutional layers and two fully connected layers. CIFAR-10 and CIFAR-100 use VGG-16 and VGG-19, respectively. The datasets and configurations used in the experiment are shown in Table 2.

The key implementation parameters are summarized in Table 3. In each update, a selected member UAV processes approximately 6% of its local samples, and the test batch size is 64. The candidate split-point set, maximum number of participating UAVs per cluster, and latency limit are also specified in Table 3. The weight parameters correspond to the energy cost, participant-selection cost, split-point cost, and joint objective defined in Equations (12), (19), (21), and (23), respectively.

The split index is defined based on the full PyTorch sequential module list, including normalization, activation and other non-trainable modules, not only convolutional and fully connected layers. For FMNIST with module sequence Conv1-BN1-ReLU-Pool1-Conv2-BN2-ReLU-Pool2-Flatten-FC1-ReLU-FC2, candidate split indices {1, 5, 9} represent boundaries after Conv1, Conv2 and Flatten. Thus, split positions are not restricted to the four trainable layers.

In each communication round, selected member UAVs upload intermediate activations after the forward pass of client-side sub-models. The cluster head executes ten consecutive server-side optimization steps on the fixed activation batch and returns activation gradients. The member UAV then performs one client-side update. Thus, one client update corresponds to ten server-side iterations.

Since different clusters can select different split points, we map sub-models back to the global model and adopt layer-wise aggregation. Let $n_{i}$, $N_{j}$ and *N* represent samples of UAV *i*, total samples of cluster *j* and global samples, respectively. The aggregation weight is $\frac{n_{i}}{N}$ for member-side layers and $\frac{N_{j}}{N}$ for cluster-head-side layers, determined by participating sample size. Split points only change the update executor, while all parameters maintain their original layer indices and tensor shapes.

### 5.2. Learning Performance Comparisons

The alternating optimization algorithm is used to update member selection and model partitioning. After receiving the intermediate activations from the selected member UAVs, each cluster head concatenates these activations and performs ten optimization steps on the cluster-head-side sub-model. These cluster-head-side steps reuse the same uploaded activations and labels; therefore, they do not introduce additional member-to-cluster-head communication or additional training samples. After the client-side and cluster-head-side updates are completed, one global aggregation is performed. The following baselines are considered. To improve comparison fairness, all methods use the same dataset partition, backbone network, data preprocessing procedure, and initial model setting whenever applicable. For each reported comparison, the same nominal member-participation ratio and global communication-round budget are used. The training-sample budget is controlled at the global-round level: each selected member UAV contributes one mini-batch in a round. For split-learning-based methods, one client-side update and the same number of cluster-head-side optimization steps are used. Method-specific operations that are intrinsic to a baseline are retained and are reported separately rather than being counted as additional communication rounds.

FedAvg: the standard federated-learning method using weighted aggregation of locally trained model parameters.

FedProx: a federated-learning method that adds a proximal term to reduce the effect of client heterogeneity.

MOON: a contrastive federated-learning method that aligns local and global representations.

SplitFed: a federated split-learning method in which clients execute the front part of the network and exchange intermediate information with the server.

SplitMix: a split-learning-based method designed for heterogeneous clients using sub-model configurations of different capacities.

ARES: a resource-aware split-learning method that adaptively selects the model split point.

To ensure comparability, all methods use the same non-IID data partitions, backbone networks, initial model parameters, preprocessing procedures, and communication-round budgets whenever applicable. The learning rates and decay factors are dataset dependent, as listed in Table 2. SGD with a momentum of 0.9 is used for optimization. In each communication round, a selected UAV processes one mini-batch containing approximately 6% of its local data. For the split-learning-based methods, each participating member performs one client-side update, and the cluster head performs ten server-side optimization steps using the uploaded activations.

FedAvg performs sample-size-weighted aggregation of the locally updated complete models. FedProx follows the same training configuration as FedAvg and introduces a proximal regularization term with a coefficient of 0.3. MOON uses the same local optimization budget and supplements the local objective with its model-contrastive loss. SplitFed uses a fixed partition in which the first two convolutional blocks are placed on the member UAV and the remaining layers are executed by the cluster head.

Because the original SplitMix method was developed for heterogeneous federated learning rather than for activation-exchange split learning, we adapt it for our experimental setting. Following the heterogeneous-model configuration used in the FedRich comparison protocol, the resource capacities of member UAVs are divided into three levels. The three groups use the first one, two, and three convolutional blocks, respectively, as their member-side sub-models, while the remaining layers constitute the cluster-head-side sub-models. The relative proportions of the three resource groups are controlled using γ=0.54369, for which $\gamma +\gamma^{2}+\gamma^{3}\approx 1$. Within the common hierarchical training architecture, the resulting member-side and cluster-head-side sub-models exchange intermediate activations and activation gradients at their respective partition boundaries. This adaptation retains the heterogeneous sub-model configuration of SplitMix while enabling its evaluation under the same activation-exchange protocol as the other split-learning-based methods.

ARES is also adapted to the common hierarchical protocol. It uses the same candidate split-point set and training budget as HCFSL-UAV, but determines the split point using its latency-energy objective with $\alpha_{ARES}=0.5$, without the joint participant-selection mechanism of HCFSL-UAV.

A nominal participation ratio of 0.2 is specified for all methods. For conventional baselines using uniform or random sampling, this corresponds to selecting 20 of the 100 available UAVs in each round. For HCFSL-UAV, however, the ratio defines the target participation budget rather than forcing exactly 20 UAVs to participate. The realized participant set is further affected by cluster association, feasibility constraints, the per-cluster participation limit, and the proposed selection criterion. Consequently, an equal nominal participation ratio does not necessarily imply equal actual participant counts or equal numbers of processed samples. The resource-cost results are calculated using the participant sets and sample counts actually realized by each method.

The key implementation settings of HCFSL-UAV are summarized in Table 3.

#### 5.2.1. Learning Accuracy Performance

Figure 3 compares test accuracy as communication rounds increase. HCFSL-UAV reaches a stable high-accuracy region earlier than the compared baselines on the three datasets and obtains the highest final test accuracy in the reported runs. The difference is relatively small on FMNIST, where all methods converge more quickly, and is more visible on CIFAR-10 and CIFAR-100, where the learning task and data heterogeneity are more demanding.

The number of runs in Table 4 refers to independent end-to-end training runs with different random seeds. These runs are used to calculate the mean and sample standard deviation of the final and best test accuracies for each dataset.

On CIFAR-10, FedAvg, FedProx, and MOON require more communication rounds to approach their final accuracy levels, while SplitFed and SplitMix exhibit noticeable fluctuations during training. ARES converges faster than these conventional baselines and achieves a final accuracy comparable to that of HCFSL-UAV. Nevertheless, HCFSL-UAV reaches a higher peak accuracy and remains competitive throughout most of the training process. On CIFAR-100, several baseline methods exhibit substantial fluctuations. ARES achieves the best overall performance among the compared baselines, whereas HCFSL-UAV follows a smoother overall improvement trend and attains the highest final accuracy among all the methods shown in Figure 3.

The observed performance of HCFSL-UAV is consistent with its joint participant-selection and adaptive model-partitioning design. The participant-selection procedure evaluates the eligible UAVs according to their current resource conditions, communication latency, and data-distribution deviation, thereby giving priority to participants that are better suited to the current training round. Adaptive model partitioning subsequently determines the split point according to the conditions of the selected UAVs. In the ARES implementation adopted for this comparison, resource-related latency and energy costs are mainly considered during split-point selection. HCFSL-UAV further coordinates this decision with participant selection within a dynamic, hierarchically aggregated UAV network.

#### 5.2.2. Training Efficiency and Resource Overhead

To evaluate training efficiency, target test accuracies of 70%, 50%, and 30% are set for FMNIST, CIFAR-10, and CIFAR-100, respectively. For each method, the required number of communication rounds is defined as the first round at which the corresponding target accuracy is reached, and the cumulative execution time up to that round is recorded. If a method does not reach the target accuracy within the predefined communication-round budget, the corresponding result is denoted as “N/R”. Figure 4 and Figure 5 compare the training efficiency, communication cost, and computation workload of the evaluated methods.

Figure 4 compares the communication rounds and cumulative execution time required by the four split-learning-based methods to reach target accuracies of 70%, 50%, and 30% on FMNIST, CIFAR-10, and CIFAR-100, respectively. The convergence round is defined as the first communication round at which the test accuracy reaches or exceeds the specified target. HCFSL-UAV generally reaches the target accuracy with fewer communication rounds and a shorter cumulative execution time than the compared methods, particularly on CIFAR-10 and CIFAR-100. The error bars represent the standard deviations across independent runs.

SplitFed and SplitMix offload part of the model computation to the server but rely on model-partitioning strategies that are not jointly adapted to the current participant set and UAV conditions. Consequently, UAVs with unfavorable computational resources or link conditions may still increase the overhead of synchronous training. ARES considers resource-related costs when determining its execution strategy, but it does not jointly coordinate participant selection and model partitioning using the coupled objective adopted by HCFSL-UAV. In contrast, HCFSL-UAV combines resource-aware participant selection with adaptive model partitioning, allowing the participating set and split point to be adjusted according to the current cluster state.

Figure 5 compares the aggregate per-round member-side communication cost and computational workload of the four split-learning-based methods. Within the reported member-side scope, HCFSL-UAV achieves the lowest mean communication and computation overhead on all three datasets. This reduction is associated with the joint adjustment of the participating set and model split point, which changes both the number of active member UAVs and the amount of computation and communication assigned to them. These results demonstrate improved member-side resource efficiency under the evaluated settings. They should not be interpreted as a complete measurement of end-to-end system resource consumption, because cluster-head computation, global aggregation, and cluster-head-to-base-station communication are not included in Figure 5.

### 5.3. Ablation Study

Ablation experiments are used to examine the separate effects of participant selection and model partitioning. Two comparison settings are considered:

Baseline A: random UAV selection within each cluster.

Baseline B: adaptive UAV selection without model-partitioning optimization.

The ablation experiments in Figure 6, Figure 7 and Figure 8 are conducted on CIFAR-10 using the VGG-16 backbone. They compare two participant-selection strategies under externally specified fixed split points: Baseline A uses random selection, whereas Baseline B uses the proposed resource- and data-aware participant-selection procedure. Neither baseline performs adaptive split-point optimization in these experiments. Therefore, Figure 6, Figure 7 and Figure 8 isolate the effect of participant selection and illustrate how the two selection strategies behave at different controlled split depths. These figures should not be interpreted as a direct evaluation of the complete HCFSL-UAV method or as decisive evidence for the advantage of the full joint-optimization framework. The complete method is evaluated separately in Figure 3, Figure 4 and Figure 5.

For the ablation analysis in Figure 8, the overall system cost is defined as an empirical evaluation metric:

(36) $$C_{sys}^{abl}=\gamma_{1}\tilde{L_{acc}}+\gamma_{2}\tilde{C_{comm}}+\gamma_{3}\tilde{C_{comp}},$$

where $\tilde{L_{acc}}$, $\tilde{C_{comm}}$, and $\tilde{C_{comp}}$ denote the min–max normalized accuracy loss, communication cost, and member-side computation workload, respectively.

Unlike the complete HCFSL-UAV algorithm, which adaptively searches the candidate set $L_{main}=\left\{1,5,9\right\}$, these experiments evaluate Baselines A and B under externally fixed split points. A denser evaluation set, $L_{abl}=\left\{2,4,6,8,10\right\}$, is used only in this ablation study to examine the effect of model-partitioning depth in greater detail. Therefore, the split points in Figure 6, Figure 7 and Figure 8 are experimental control values rather than the candidate set searched by the complete HCFSL-UAV algorithm.

#### 5.3.1. Effect of Split Point on Performance

Figure 6 shows that test accuracy varies with the externally fixed split point. This variation should not be attributed solely to the increase in member-side computation associated with a deeper split. Under exact end-to-end optimization with an unchanged participant set and training budget, repartitioning the same network mainly redistributes computation between the member UAV and the cluster head and does not necessarily reduce model accuracy. In the present experiments, however, changing the split point also changes member-side feasibility, the realized participating set, the number and distribution of processed samples, the location at which activations and gradients are exchanged, and the relative update behavior of the member-side and cluster-head-side sub-models. The observed accuracy reduction at deeper split points is therefore more appropriately interpreted as the combined effect of these changes rather than as a direct consequence of increased computational workload alone.

#### 5.3.2. Communication and Computation Cost Analysis

Figure 7 reports the realized aggregate member-side communication and computation costs of Baselines A and B. Because the participant-selection rules may produce different participant and sample counts, the differences shown in Figure 7 do not measure the isolated effect of the split point alone. Instead, they reflect the combined effects of the fixed split location and the participant set produced by each selection strategy. If the participating UAVs and processed-sample counts were held identical, the two baselines would have the same raw member-side communication and computation costs at a given split point.

#### 5.3.3. Overall System Cost

Figure 8 summarizes the trade-off among normalized accuracy loss, member-side communication cost, and member-side computation cost. Baseline B achieves a lower system-cost value over the evaluated fixed split points, indicating that the proposed participant-selection strategy provides a more favorable trade-off than random selection under these controlled settings. This result supports the effectiveness of the participant-selection component.

The figure therefore supports two observations used in the HCFSL-UAV design: participant selection has a measurable effect on system cost, and the preferred split point depends on the communication–computation trade-off. These observations motivate optimizing the two decisions jointly rather than fixing either one in advance.

### 5.4. Greedy Optimality and Runtime Analysis

To evaluate the trade-off between solution quality and computational complexity, the greedy participant-selection method was compared with exhaustive search on small-scale problem instances for which the globally optimal solution could be obtained. Each instance contained 10 candidate UAVs, the maximum number of selected UAVs was five, and the candidate split-point set was {1, 5, 9}. Ten independent instances were evaluated. These ten instances are independently generated, small-scale combinatorial optimization problems used only to compare the greedy participant-selection procedure with exhaustive search. They are not neural-network training runs and are independent of the dataset-specific repetitions reported in Table 4. The number of instances is limited to ten because exhaustive enumeration is computationally expensive even at this reduced problem scale.

The optimality gap was calculated as

(37) $$Gap=\frac{\left(J_{\mathrm{greedy}}-J_{\mathrm{opt}}\right)}{J_{\mathrm{opt}}}\times 100\%$$

where $J_{greedy}$ and $J_{opt}$ denote the objective values obtained by the proposed greedy method and exhaustive search, respectively.

The greedy method produced a mean optimality gap of 17.42%, with a standard deviation of 27.01% and a median gap of 2.74%. It obtained the exact optimal solution in two of the ten instances. The mean execution times of the greedy method and exhaustive search were 3.40 ms and 64.44 ms, respectively, corresponding to an average runtime speedup of approximately 18.95 times. The difference between the mean and median gaps indicates that the greedy method achieves a relatively small gap in most tested instances but may produce a larger gap in unfavorable cases. Therefore, the proposed algorithm is regarded as a computationally efficient approximation rather than a globally optimal solution. Its main advantage is that it avoids the exponential complexity of exhaustive participant-set enumeration.

### 5.5. Summary of Ablation Results

The ablation results show that adaptive UAV selection improves the quality of the participating set and reduces unnecessary resource consumption relative to random selection. They also show that changing the split point shifts cost between communication and member-side computation. The two mechanisms address different parts of the training cost and are therefore treated as coupled decisions in the proposed framework.

## 6. Conclusions

This paper studies distributed learning in UAV-assisted edge-intelligence systems with resource constraints, dynamic network conditions, and non-IID data. HCFSL-UAV is proposed as a three-tier federated split-learning framework composed of a central base station, cluster-head UAVs, and member UAVs. Member UAVs execute the front part of the model, cluster heads complete the remaining computation and perform intra-cluster aggregation, and the central base station performs global aggregation.

The framework combines dynamic member-UAV selection with adaptive model partitioning. Member selection considers resource feasibility and data representativeness, while model partitioning adjusts the split point according to the selected UAV set. A joint optimization model is formulated using data-distribution deviation, training latency, and resource cost, and an alternating optimization procedure is used to update the two discrete decisions through greedy member selection and split-point search.

Experiments on FMNIST, CIFAR-10, and CIFAR-100 show that HCFSL-UAV reaches higher final test accuracy and faster convergence than the compared methods in the reported settings. The split-learning comparisons also show lower per-round communication cost and computation workload for HCFSL-UAV. The ablation results further show that adaptive participant selection reduces cost relative to random selection and that the split point changes the balance between communication and local computation.

Future work will jointly consider UAV trajectories, cluster-head association, and bandwidth allocation under changing channel conditions. The scalability of the method will be examined in larger UAV networks, together with the sensitivity of its performance to the objective-function weights. Privacy-preserving reporting of the label statistics used for participant selection will also be investigated.

## Figures and Tables

**Figure 1:**
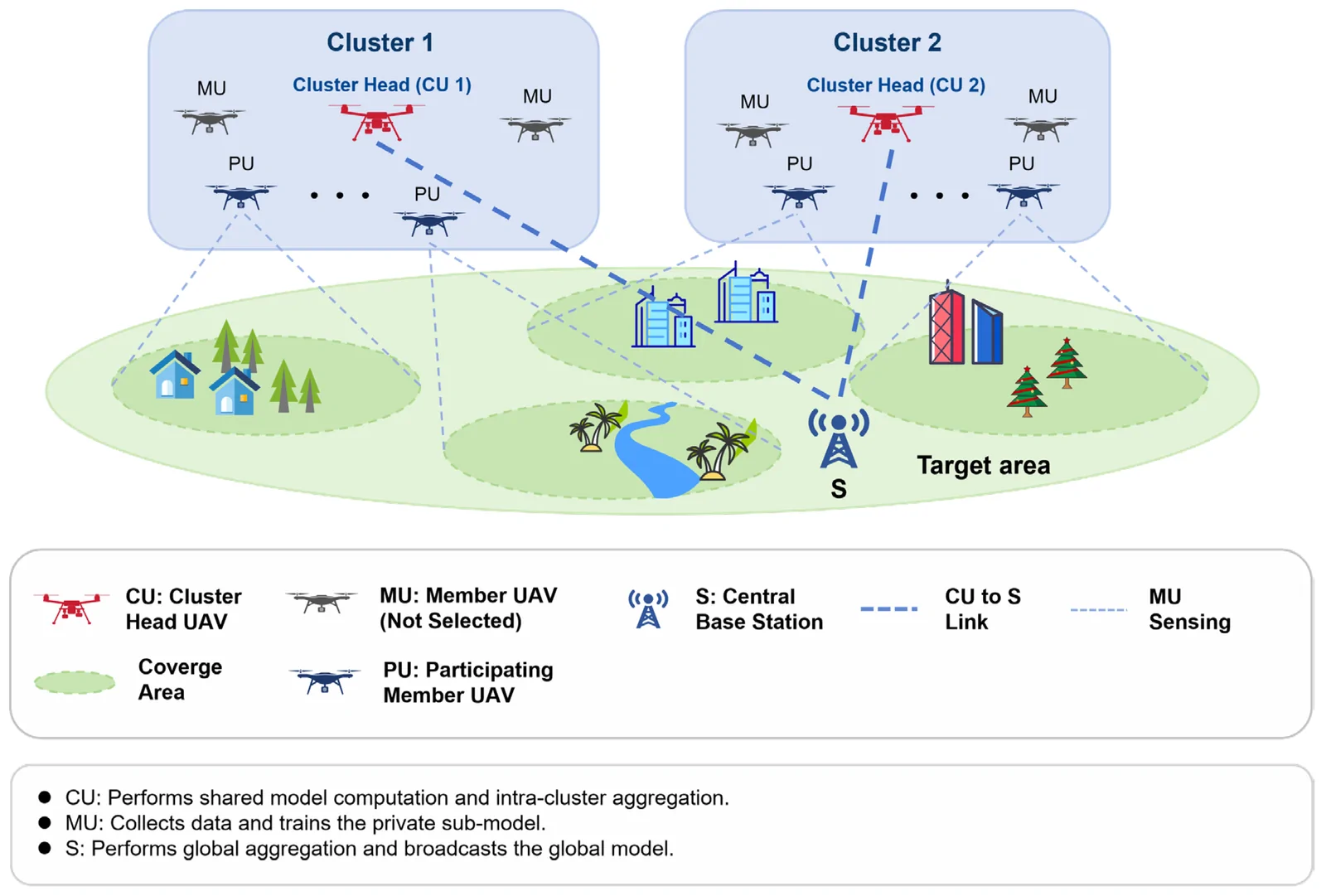
System model.

**Figure 2:**
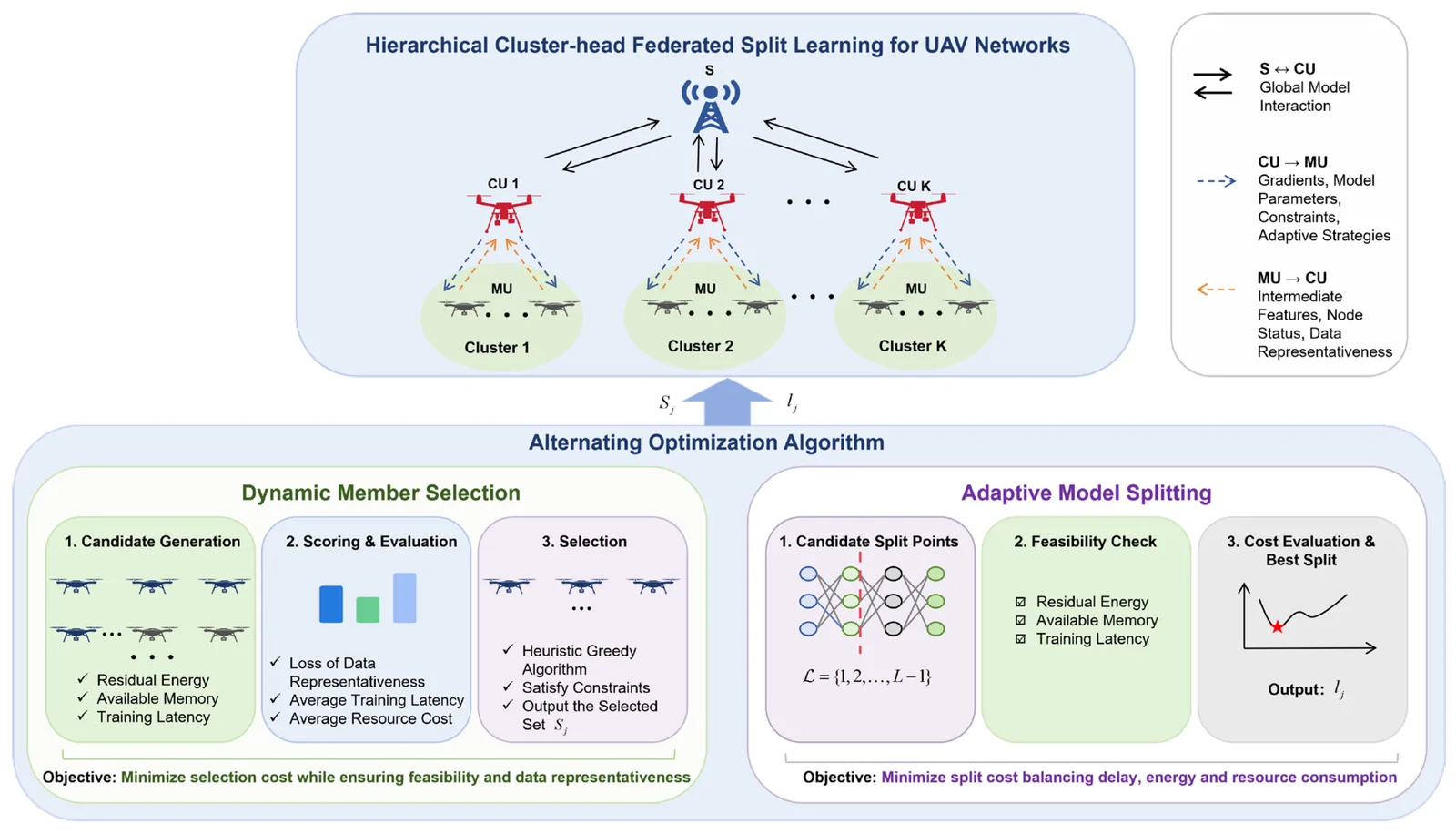
Hierarchical cluster-head federated split learning framework for UAV Networks.

**Figure 3:**
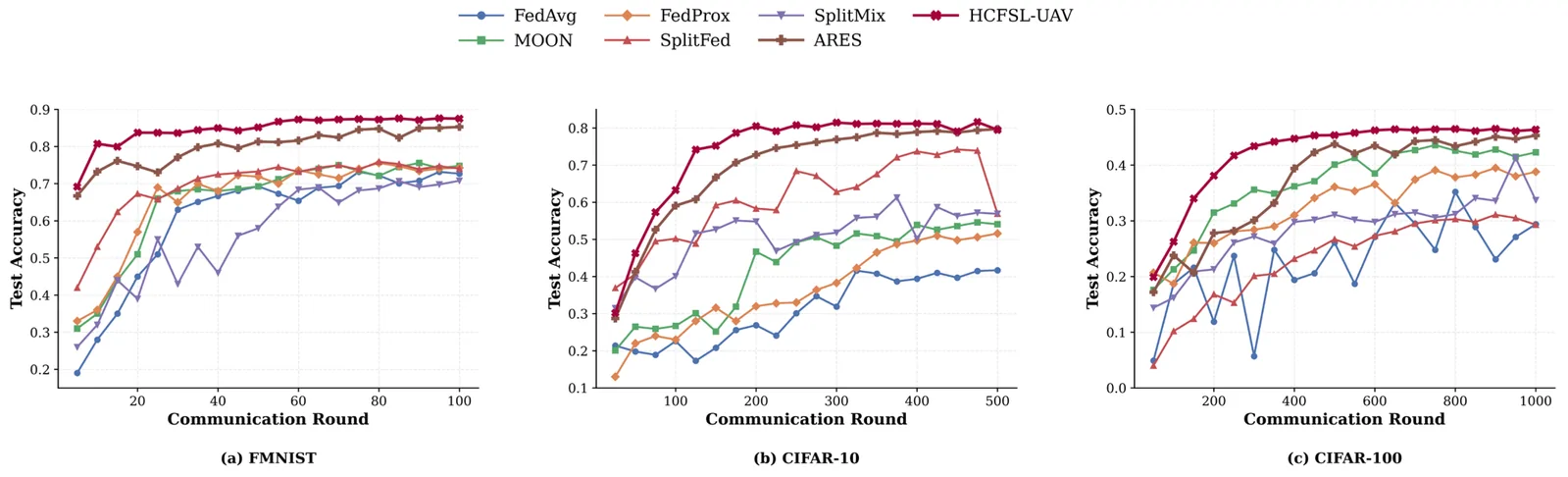
Test accuracy versus communication rounds on different datasets.

**Figure 4:**
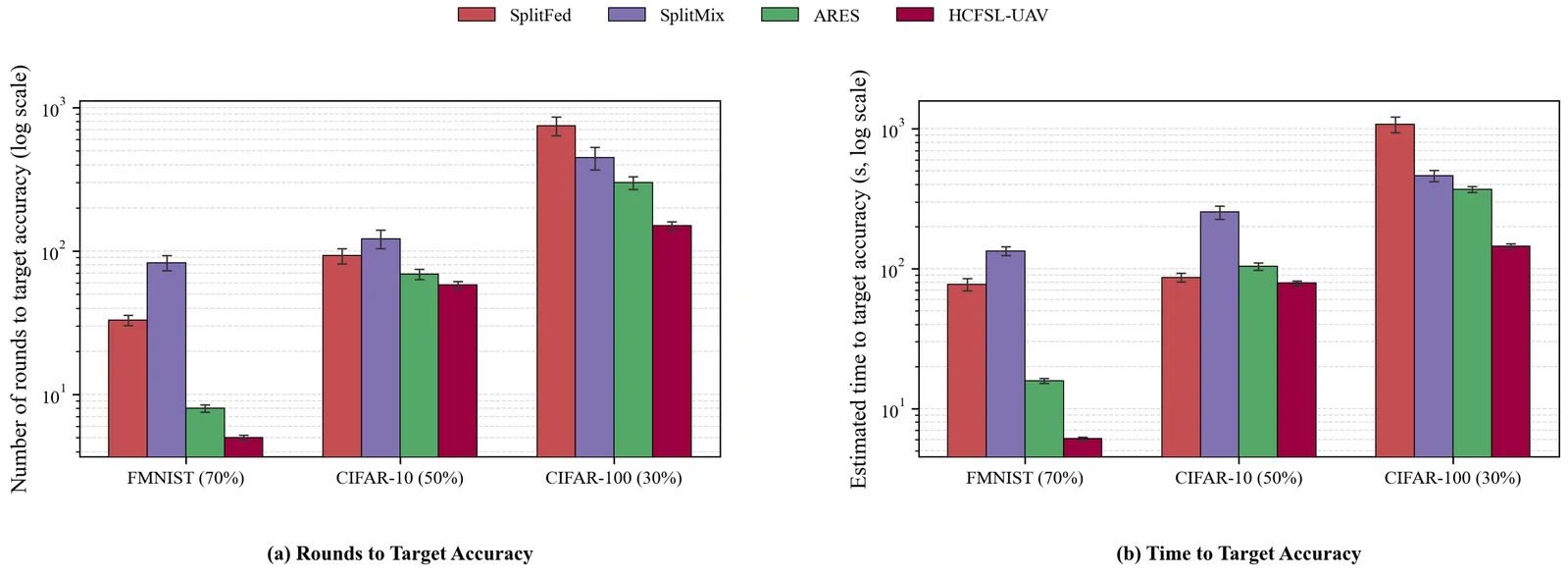
Communication rounds and estimated cumulative execution time required by the selected split-learning-based methods to reach target accuracies of 70%, 50%, and 30% on FMNIST, CIFAR-10, and CIFAR-100, respectively. The vertical axes are plotted on logarithmic scales. Error bars represent the standard deviations across independent runs.

**Figure 5:**
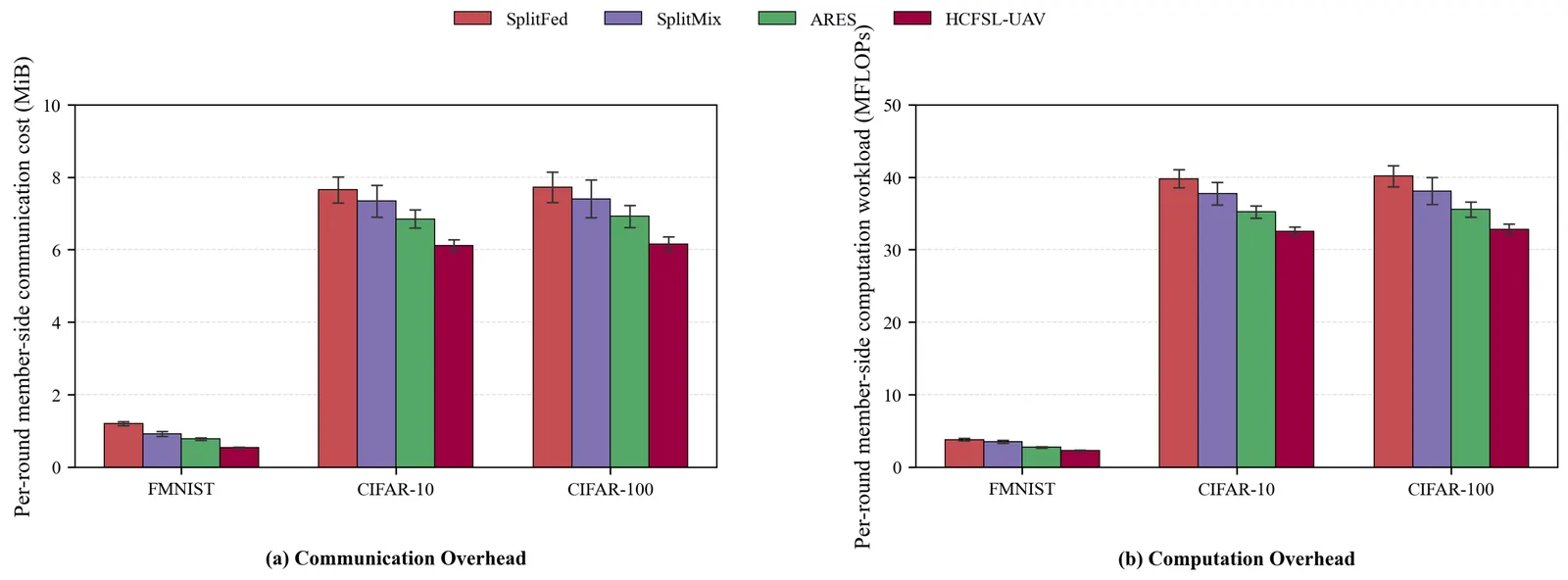
Per-round member-side overhead of the split-learning-based methods on FMNIST, CIFAR-10, and CIFAR-100: (**a**) communication cost and (**b**) computational workload. Bars and error bars represent the mean and standard deviation across independent runs, respectively. Only the communication and computation performed by member UAVs are included; the cluster-head-side workload is excluded.

**Figure 6:**
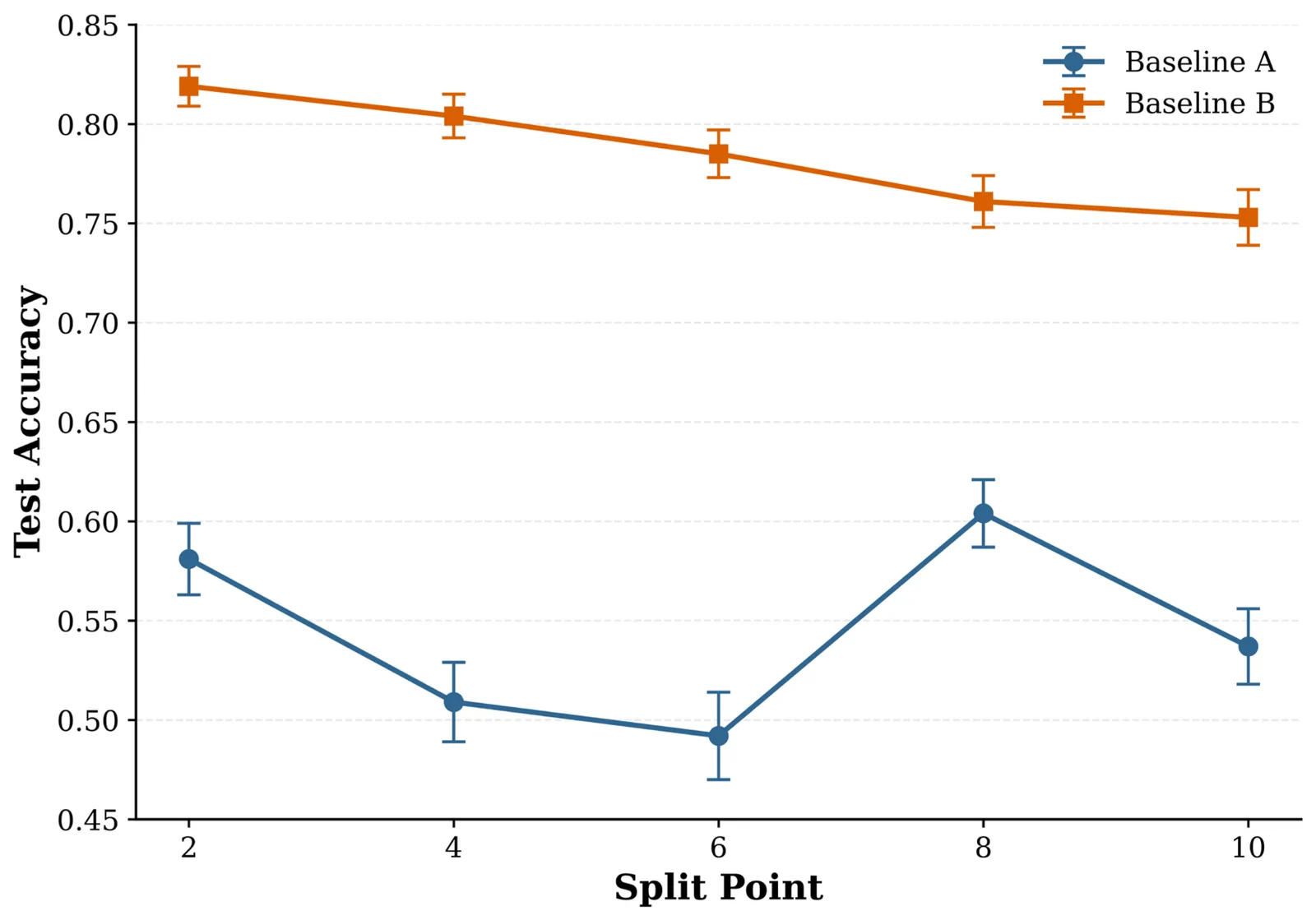
Test accuracy of Baselines A and B under different fixed split points on CIFAR-10 using VGG-16. Error bars denote the standard deviations across independent runs.

**Figure 7:**
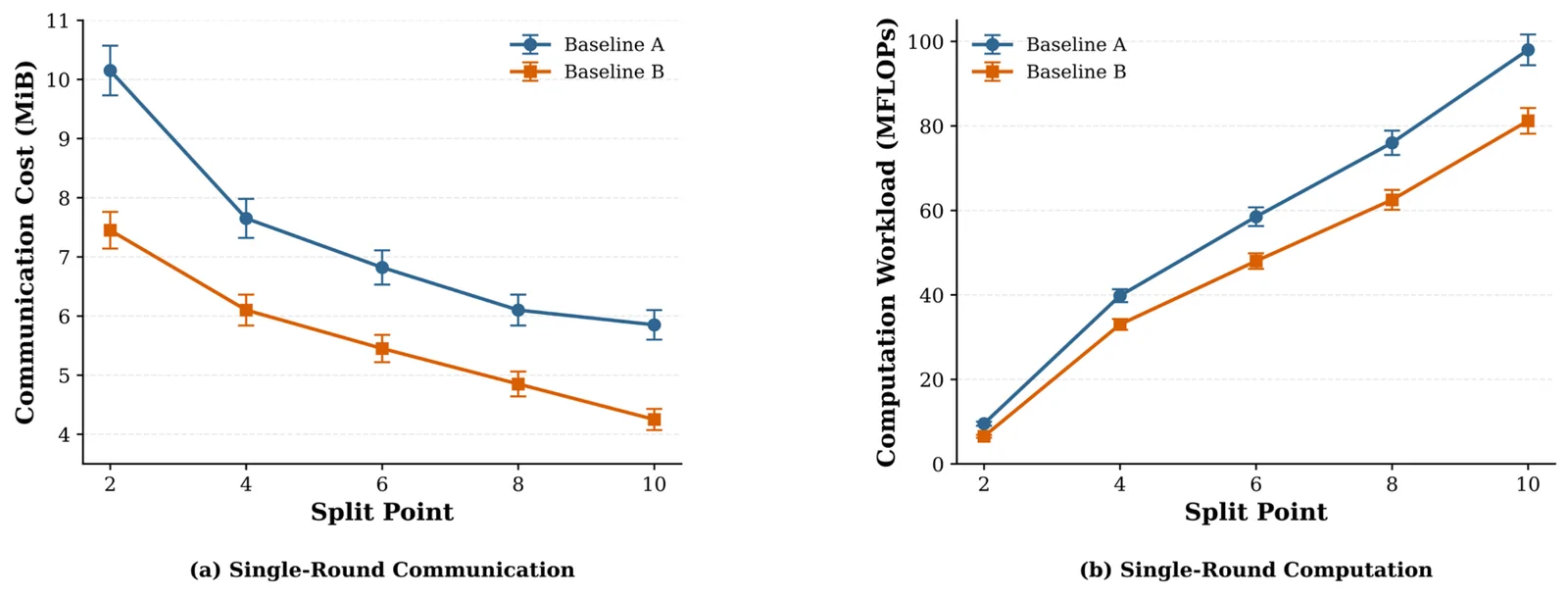
Per-round communication cost and computational workload of Baselines A and B under different fixed split points on CIFAR-10 using VGG-16. Error bars denote the standard deviations across independent runs.

**Figure 8:**
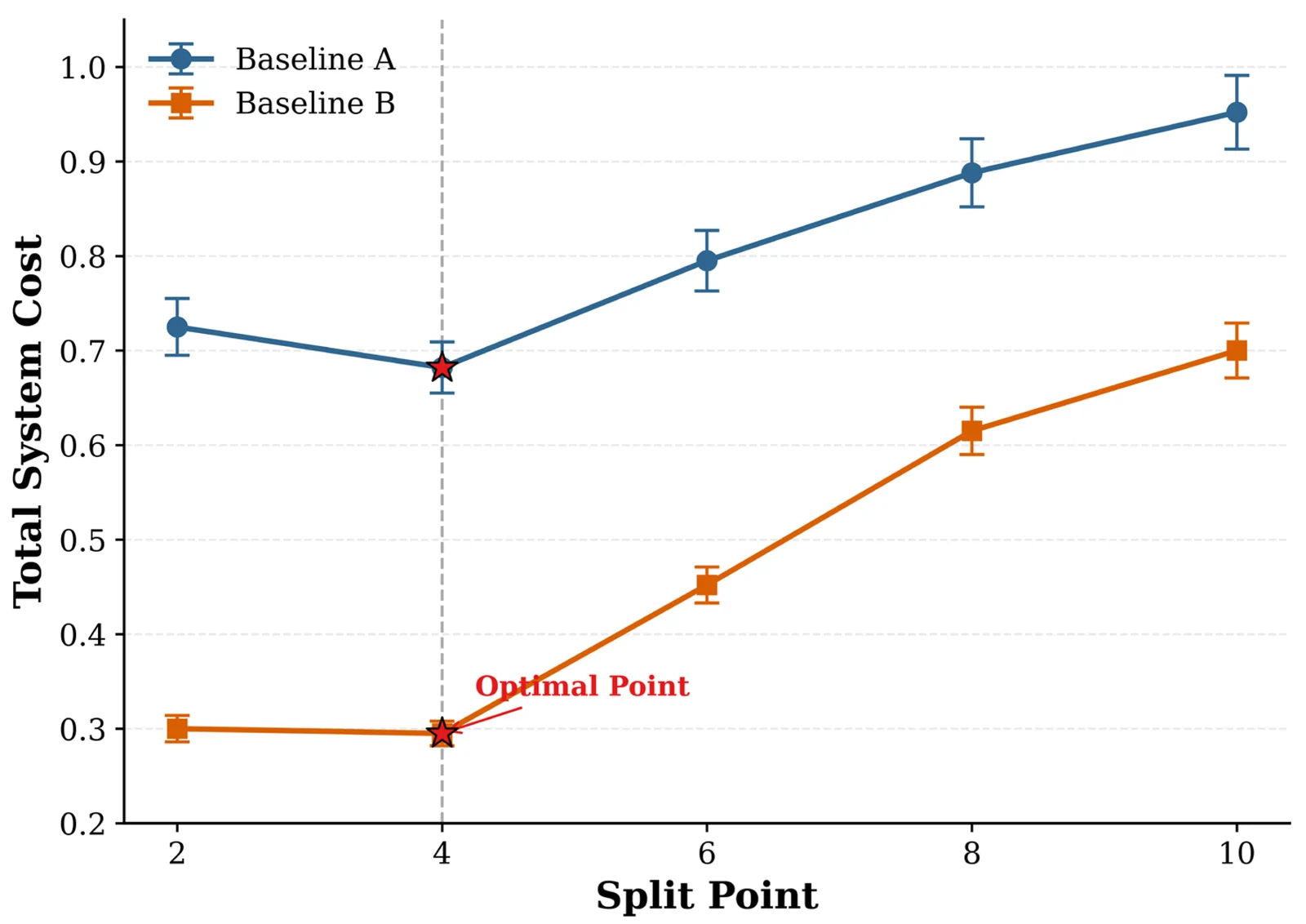
Overall system cost of Baselines A and B under different fixed split points on CIFAR-10 using VGG-16. Error bars denote the standard deviations across independent runs.

**Table 1:** Simulation parameters of the wireless UAV network.

Parameter	Symbol	Value	Unit
Uplink bandwidth	$W_{ij}^{UL}$	10	MHz
Downlink bandwidth	$W_{ij}^{DL}$	10	MHz
Noise power spectral density	$N_{0}$	−174	dBm/Hz
CPU frequency of member UAV *i*	$f_{i}$	$0.5\times 10^{9}$	cycles/s
Computation capability per CPU cycle	$\rho_{i}$	1	FLOP/cycle
Uplink transmit power of member UAV *i*	$p_{i}^{UL}$	[0.1, 0.3]	W
Downlink transmit power allocated by cluster−head UAV *j*	$p_{ij}^{DL}$	[0.4, 0.6]	W
Receiving power of member UAV *i*	$p_{i}^{RX}$	[0.5, 0.15]	W
Path-loss exponent	*η*	2.2	-
Reference channel power gain at 1 m	$\beta_{0}$	−30	dB
Rician factor	$K_{Ric}$	10	dB
Duration of one communication round	ΔT	1	s
Initial residual energy	$E_{i}(0)$	[500, 700]	J
Residual-energy threshold	$E_{min}$	50	J
Available memory	$M_{i}^{ava}$	[1024, 2048]	MB
Maximum latency	$T_{max}$	5	s

**Table 2:** Datasets and configurations used in the experiment.

Dataset	Model	Learning Rate	Decay Rate	Number of Classes per Client
FMNIST	2 conv + 2 fc	0.1	0.98	2
CIFAR-10	VGG-16	0.03	0.995	2
CIFAR-100	VGG-19	0.03	0.996	20

**Table 3:** Key implementation and optimization parameters of HCFSL-UAV.

Parameter	Symbol	Value
Candidate split points in the main experiments	$L_{main}$	{1,5,9}
Fixed split points in the ablation experiments	$L_{abl}$	{2,4,6,8,10}
Nominal local mini-batch size	$B_{i}$	$\max\{1,round(0.06n_{i})\}$
Test batch size	-	64
Maximum UAVs per cluster	$N_{j}^{max}$	10
Participant-selection weights, Equation (19)	$\left(\omega_{1},\omega_{2},\omega_{3}\right)$	(0.60,0.20,0.20)
Split-point weights, Equation (21)	$\left(\mu_{1},\mu_{2},\mu_{3}\right)$	(0.50,0.25,0.25)
Joint-objective weight, Equation (23)	α	0.50
Computation/communication energy weights, Equation (12)	$\left(\lambda_{1},\lambda_{2}\right)$	(0.50,0.50)
Ablation evaluation weights, Figure 8	$\left(\gamma_{1},\gamma_{2},\gamma_{3}\right)$	$\left(\frac{1}{3},\frac{1}{3},\frac{1}{3}\right)$
Minimum participating UAVs per cluster	$N_{j}^{min}$	1
Maximum AO iterations	$R_{max}$	5
AO stopping tolerance	ε	$10^{-3}$

**Table 4:** Final and best test accuracies of HCFSL-UAV on three datasets.

Dataset/Method	Runs	Final Accuracy (%)	Best Accuracy (%)
FMNIST/HCFSL-UAV	5	85.782±1.051	86.428±0.243
CIFAR-10/HCFSL-UAV	3	80.391±0.737	81.934±0.802
CIFAR-100/HCFSL-UAV	3	45.515±0.377	46.056±0.668

## Data Availability

The datasets FMNIST, CIFAR-10 and CIFAR-100 are publicly available. Further inquiries can be directed to the corresponding author.
